# Special Issue “Hematopoietic System Under Physiological Conditions and Following Hematopoietic Reconstitution or Stress: Second Edition”

**DOI:** 10.3390/ijms262110603

**Published:** 2025-10-31

**Authors:** Maria Kalashnikova, Alexander Belyavsky

**Affiliations:** Engelhardt Institute of Molecular Biology, Russian Academy of Sciences, Vavilova 32, 119991 Moscow, Russia; alopexmary@gmail.com

The hematopoietic system is one of the most pervasive systems in mammalian organisms and is uniquely positioned for the integration of the body’s various organs. Hematopoietic stem and progenitor cells (HSPCs) are the major drivers of hematopoiesis and are responsible for producing enormous numbers of mature effector cells. To ensure the proper homeostatic functioning of hematopoietic system throughout the organism’s life cycle, HSPCs must maintain genome integrity and balance self-renewal with differentiation decisions, coordinating these with both the organism’s normal functions and its responses to environmental stresses and various infectious challenges. To decipher the fundamental aspects of the hematopoietic system, current research integrates advanced molecular, genetic, cellular, and bioinformatics techniques to enable comprehensive analysis of both HSPCs and bone marrow niche in their previously unrecognized complexity. This Special Issue was designed to raise awareness among biomedical specialists of the intricacies of HSPC biology and its complex interactions with bone marrow niches. It includes four experimental [1,2,3,4] and one review article [5] reporting state-of the-art findings and recent progress in this prominent field of biomedicine.

Ex vivo expansion represents a major challenge for various advanced clinical applications of HSPCs. An important study by Zehnle et al. [1] addresses with effects of apoptosis inhibition during HSPC ex vivo expansion on their reconstitution potential. It has been shown previously that ex vivo cultured HSPCs are markedly prone to apoptosis [6]. Moreover, depletion of pro-apoptotic proteins Bim and Bmf significantly enhances early engraftment and long-term reconstitution of HSPCs [7]. In the study by Zehnle at al., ex vivo expansion of human CD34^+^ umbilical cord blood (UCB) cells was performed using SCF, TPO, FLT3L and IL-6 in combination with the aryl hydrocarbon receptor antagonist StemRegenin 1 (SR-1) [8]. The observed expansion of total and more primitive CD34^+^CD38^-^ cells was accompanied by a gradual decrease in cell viability, while SR-1 had little effect on either expansion or viability. The authors then tested the effects of lentivirus-mediated expression of the anti-apoptotic protein BCL-X_L_ on cell expansion. In the bicistronic lentivector used, BCL-X_L_ expression was coupled to that of EGFP, enabling the monitoring of cells expressing BCL-X_L_. Cells transduced with control vector lacking BCL-X_L_ demonstrated a steady decline during expansion, indicating that lentiviral transduction substantially reduced cell fitness. In contrast, the proportion of BCL-X_L_-expressing cells remained stable at a much higher level. The fraction and total number of EGFP^+^CD34^+^ cells were also increased in the BCL-X_L_-transduced group. The authors further demonstrated that BCL-X_L_ expression enhances the repopulation potential of ex vivo expanded HSPCs using xenotransplantation of the expanded cells into Rag2^−/−^γc^−/−^ mice. Importantly, engraftment of lentivirally transduced cells in the bone marrow two months post-transplantation was only observed when BCL-X_L_ was overexpressed. The same pattern was found in the spleen and thymus. The authors also analyzed the effects of pan-caspase inhibitor Q-VD-OPh and necroptosis inhibitor necrostatin 1 during ex vivo expansion phase, but no beneficial effects were revealed. In summary, this study demonstrates that lentiviral transduction markedly decreases the survival and engraftment of human HSPCs, whereas BCL-X_L_ overexpression exerts unequivocal positive effects on HSPCs and neutralizes the negative effects of transduction. Contrary to previous reports [9] and of importance for clinical perspective, the presence of SR-1 during the expansion phase did not provide demonstratable benefits.

It is now well established that definitive HSCs in mammals originate during embryogenesis from specialized “hemogenic endothelium” in the aorta and major arteries through a process known as the endothelial-to-hematopoietic transition (EHT). During EHT, hemogenic endothelial cells (HECs) destined to become HSCs bud from the arterial endothelial wall, migrate to the fetal liver, and finally colonize the bone marrow. Despite significant progress in deciphering the molecular mechanism underlying EHT, this field remains largely underexplored. In the work by Shalaby et al. [2], the authors exploited existing single-cell transcriptomics data [10] from the human embryo AGM region to identify signaling pathways involved in EHT that may influence the formation of nascent HSCs. To extract additional information from the single-cell data, they used a recently developed bioinformatics method [11] and identified, in addition to HECs, HSCs, and mature hematopoietic cells, stromal and endothelial cell populations. Their analysis revealed 142 unique signaling pathways in the AGM microenvironment acting on HECs. Importantly, several of these pathways were previously shown to support EHT, including TGFβ, Notch, and Wnt signaling modules [12,13,14]. The authors also identified several receptor–extracellular matrix interactions highly specific to HECs. Other findings included confirmation of the role of sterile inflammation in HSC specification during EHT, with a specific involvement of TNFR2 and STAT3. The roles of Osteopontin and matrix metalloproteinases in the release of HSC from the arterial endothelium was also revealed. Finally, the authors identified potential interactions between HECs and the fetal liver that may enhance the release of nascent HSCs from endothelium into circulation. In summary, using advanced bioinformatics tools, the authors explored complex, inter-regulated signals among niche cells, circulating hematopoietic cells, and the distal fetal liver. They identified a number of signaling pathways potentially active in the EHT process, some of which were previously characterized and others newly proposed, warranting future experimental validation.

An important study by Bruno et al. [3] addresses the organism’s response to infection. It is now well known that severe infections induce HSPCs to exit quiescence and that infections exert significant and long-lasting effects on HSPCs [15,16,17]. However, these effects are mostly studied in acute disease models, and the potential for functional recovery of the HSPC compartment following infection remains largely unexplored. In particular, previous studies using a mouse model of severe malaria caused by *Plasmodium berghei* demonstrated induction of emergency myelopoiesis accompanied by reduced erythroid and lymphoid cell production, as well as a loss of phenotypic and functional HSCs [18,19]. However, because *Plasmodium berghei* infection is fatal in mice, it cannot provide information about recovery from infection. In their study, Bruno et al. used a model of natural rodent malaria induced by *Plasmodium chabaudi*, which produces a self-resolving disease, to investigate the effects of such infection on the HSPC compartment. The authors first characterized the time course of infection and determined the optimal time points for sample collection. They further demonstrated that the peak of parasitemia occurred at day 11 paralleled by the development of anemia due to RBC loss at the same time point, followed by gradual RBC restoration to normal levels by day 29. The major cytokines induced during infection were IFN-γ and TFN-α, both peaking around day 11. Importantly, studies of BM composition at different time points revealed an approximately two-fold reduction in a number of phenotypically most primitive quiescent CD150^+^CD48^-^ LKS stem cells at days 11 and 15, followed by a highly variable rebound phase and restoration of normal levels by day 60. At the same time, the levels of myeloid-primed MPP2 and MPP3 populations increased dramatically by 40- to 50-fold, at day 11, with a return to baseline by day 24. In contrast, lymphoid-primed MPP4 populations demonstrated a smaller, 6-fold increase at the peak of infection. The analysis of the proliferation kinetics of different HSPC subsets using EdU incorporation confirmed these results, revealing a pronounced increase in the absolute numbers of proliferating cells within the MPP2–4 populations. In contrast to the strong expansion of multipotential cell populations, the committed progenitor populations, namely common myeloid progenitors (CMPs), granulocyte–macrophage progenitors (GMPs), megakaryocyte–erythrocyte progenitors (MEPs) and common lymphoid progenitors (CLPs), demonstrated a drastic decrease at the peak of infection. Overall, the various HSPC and committed progenitor populations showed strong, highly dynamic, and subset-specific responses to *Plasmodium chabaudi* infection, gradually returning to normal between days 24 and 60 depending on the cell subset.

An interesting study by Borges et al. [4] focused on a topic highly relevant to both basic and clinical research, namely polycythemia vera (PV), the most common type of Philadelphia chromosome-negative myeloproliferative disease. PV is a clonal disorder caused in most cases by activating mutations in the JAK2 kinase gene [20]. Although it is generally manageable with modern therapeutics and thus relatively benign, PV leads to excessive thrombocytic, erythroid, and myeloid cell production, which might lead to thrombosis and fatal outcomes if not adequately treated [21]. Disturbances of blood cell homeostasis in PV manifest, among other effects, as maladaptive stress erythropoiesis leading to autonomous, cytokine-independent erythroid cell proliferation [22]. The article by Borges et al. describes the results of experiments investigating the potential role of blood monocytes in clearing erythrocytes in PV vs. healthy individuals. The authors observed alterations in specific monocyte subsets in PV and demonstrated that PV erythrocytes are likely to be more susceptible to monocyte clearance due to reduced expression of SIRP-a on PV monocytes, a finding consistent with previous reports [23]. Importantly, the was a more than 10-fold increase in the percentage of glycophorin A-positive monocytes in PV patients compared to healthy donors. Glycophorin A levels were also substantially higher in PV monocytes than in normal monocytes. These findings provide strong evidence that during PV, monocytes become more active in erythrocyte clearance. In addition, in vitro experiments demonstrated that PV erythrocytes also contribute to enhanced clearance. PV monocytes more efficiently phagocyted both normal and PV RBCs, and PV RBC-derived material was more efficiently taken up by monocytes, regardless of their origin. In addition to these crucial findings, the authors also showed that PV monocytes exhibited higher expression of erythroid cell adhesion molecules CD169, CD163, and VCAM-1, suggesting potential interference with erythropoiesis. Overall, the results of this study may be pointing to existence of compensatory mechanisms in PV, particularly enhanced erythrocyte clearing in the disease state.

The review article by Watt and Roubelakis, comprising more than 400 references [5], represents a very thorough and wide-ranging analysis of current state-of-the-art in human HSPC biomedical research. This review is both timely and important, as most existing knowledge about the hematopoietic system has been derived from mouse models, which differ substantially from the human system.

The authors first provide a concise overview of milestones achieved in human HSC research and transplantation practice, from the identification of bone marrow as the primary source of blood cells in the 19th century to the first successful use of HCT for treating primary immunodeficiency disorders in 1968.

They then describe the evolution of the HSC niche concept, particularly in relation to the development of CFU-S assays and in vitro culture systems as surrogate assays for HSCs. This is followed by an overview of murine xenograft models used for analyzing primitive human HSPCs with repopulating or self-renewal potential. The review also discusses steady advances in human hematopoietic cell transplantation, the development of alternative HSC sources for clinical use, and progress in identifying and optimizing factors contributing to increasing clinical success of this therapeutic avenue.

In the next section, the authors examine the daily turnover of hematopoietic cells under steady-state conditions in humans, followed by a discussion of the hallmarks of stemness in human HSCs and analyses of their potency and differentiation trajectories. They highlight key challenges in decoding the intricacies of human hematopoiesis. In particular, CD34 and CD133 serve as important markers of human HSCs with reconstitution capacity, but cell populations defined by them are highly heterogeneous, with true HSCs constituting only a small minority subset. Subsequent sections discuss major experimental findings related to CD34 and CD133 as key HSPC biomarkers, as well as additional surface markers used to further characterize HSPC subsets, including CD38, CD33, CD201 and CD90, alongside emerging discoveries based on their application.

The authors further address models of human hematopoiesis starting with the classical hierarchical model. These newer models postulate a continuum of lympho-myeloid progenitors downstream of HSCs, suggesting far greater flexibility in differentiation decisions that was previously envisioned. Two evolving models are discussed in detail: the continuum model, in which low-primed HSPCs directly differentiate into unilineage progenitors, and the punctuated continuum model, characterized by hierarchically structured transition points.

Next, the authors explore the sophisticated approaches, in particular barcoding, to lineage tracing of HSPCs and progeny dynamics in surrogate models, noting differences in outcomes depending on whether analysis was performed under steady-state or post-myeloablative conditions. They further review the application of lineage tracing approaches for human hematopoiesis, including the analysis of “endogenous barcodes” such as germline and somatic nuclear and mitochondrial variants.

The review also examines heterogeneity and potential inequality among bone marrow anatomical sites in terms of hematopoiesis support in mice and humans, particularly in response to hematopoietic stress and injury. In addition, the authors address the issue of extramedullary sites participation in normal hematopoiesis and summarize recent key findings in this field.

Later sections summarize discoveries related to long-term and short-term repopulating cells and MPPs, as well as the identification of deeply quiescent and less quiescent murine HSC subsets. The authors then discuss an important and currently controversial debate surrounding the contribution of dormant HSCs to steady-state hematopoiesis and their response to severe stress or sepsis. They also consider the similarly controversial topic of the co-existence of balanced multilineage HSCs and those biased in particular lineage, including platelet-biased, myeloid-biased, or lymphoid-biased HSCs.

In the final part of their review, the authors address an increasingly important topic, namely the changes in hematopoietic system during aging. They describe the most salient features of the aging hematopoiesis, in particular the myeloid bias and an increased number of phenotypically defined HSCs with reduced functional capacity, as well as more subtle changes occurring in the aging hematopoietic system. The authors also describe the recently discovery of innate immunological memory, which leads to epigenetic reprogramming of HSPCs, resulting in either enhanced (trained immunity) or suppressed (immune tolerance) secondary response to repeated pathogen exposure. Finally, the authors discuss the critical role of metabolic regulation, particularly involving mitochondria and proteostasis, in HSC homeostasis and developmental decisions.

In summary, this Special Issue encompasses a wide spectrum of topics related to hematopoiesis research, featuring state-of-the-art original studies, as well as a comprehensive and highly informative review on human hematopoiesis. We believe that these contributions will be of substantial interest both to professionals working in this field and to the biomedical audience in general.
